# Broaden Horizons: The Advancement of Interstitial Cystitis/Bladder Pain Syndrome

**DOI:** 10.3390/ijms232314594

**Published:** 2022-11-23

**Authors:** Jin Li, Xianyanling Yi, Jianzhong Ai

**Affiliations:** 1 West China School of Medicine, Sichuan University, Chengdu 610041, China; 2 Department of Urology, Institute of Urology, Sichuan University, Chengdu 610041, China

**Keywords:** interstitial cystitis, pain, bladder, treatment, diagnosis

## Abstract

Interstitial cystitis/bladder pain syndrome (IC/BPS) is a debilitating disease that induces mental stress, lower urinary symptoms, and pelvic pain, therefore resulting in a decline in quality of life. The present diagnoses and treatments still lead to unsatisfactory outcomes, and novel diagnostic and therapeutic modalities are needed. Although our understanding of the etiology and pathophysiology of IC/BPS is growing, the altered permeability of the impaired urothelium, the sensitized nerves on the bladder wall, and the chronic or intermittent sensory pain with inaccurate location, as well as pathologic angiogenesis, fibrosis, and Hunner lesions, all act as barriers to better diagnoses and treatments. This study aimed to summarize the comprehensive information on IC/BPS research, thereby promoting the progress of IC/BPS in the aspects of diagnosis, treatment, and prognosis. According to diverse international guidelines, the etiology of IC/BPS is associated with multiple factors, while the presence of Hunner lesions could largely distinguish the pathology, diagnosis, and treatment of non-Hunner lesions in IC/BPS patients. On the basis of the diagnosis of exclusion, the diverse present diagnostic and therapeutic procedures are undergoing a transition from a single approach to multimodal strategies targeting different potential phenotypes recommended by different guidelines. Investigations into the mechanisms involved in urinary symptoms, pain sensation, and bladder fibrosis indicate the pathophysiology of IC/BPS for further potential strategies, both in diagnosis and treatment. An overview of IC/BPS in terms of epidemiology, etiology, pathology, diagnosis, treatment, and fundamental research is provided with the latest evidence. On the basis of shared decision-making, a multimodal strategy of diagnosis and treatment targeting potential phenotypes for individual patients with IC/BPS would be of great benefit for the entire process of management. The complexity and emerging evidence on IC/BPS elicit more relevant studies and research and could optimize the management of IC/BPS patients.

## 1. Introduction

Defined as a chronic inflammatory condition with abacterial infections of the bladder, interstitial cystitis/bladder pain syndrome (IC/BPS) is characterized by symptomatic frequency and urgency, as well as chronic pelvic pain [1,2,3,4]. Although non-lethal, IC/BPS afflicts millions of individuals worldwide, while its potential mechanism is unknown. Many studies have gradually launched investigations into the etiology and pathophysiology of IC/BPS. It is noteworthy that Hunner lesions were observed in a proportion of IC/BPS patients, which was an indicator of a better prognosis after lesion-targeted therapy compared to non-Hunner lesions IC/BPS (NHIC) [5,6]. This evidence inspired phenotypic stratifications in IC/BPS patients, and the classification systems were highly recommended, as suggested in the American Urological Association (AUA) [2,7], the Canadian Urological Association (CUA) [8] and the European Association of Urology (EAU) [3]. Furthermore, the UPOINT [9] (urinary, psychosocial, organ-specific, infection, neurologic/systemic, and tenderness) and INPUT [10] (infection, neurologic/systemic, psychosocial, ulcers, and tenderness of muscles) classifications have emerged as novel phenotypic systems for the optimization of the treatment modalities, but their effectiveness still requires further exploration. On this foundation, several guidelines recommend various treatment algorithms with modalities solely or jointly in the treatment of IC/BPS patients in different stages [1,2,3,4,8,11,12]. Additionally, novel delivery systems [13,14,15,16,17], monoclonal T antibodies [18,19,20,21], stem cell therapies [22,23], and other innovative therapies also emerge as options for patients with newly diagnosed or refractory IC/BPS. However, the efficacy of IC/BPS treatment remains unsatisfactory, despite the present and emerging therapies. 

Moreover, IC/BPS is a disorder that requires the active participation of patients. It is imperative for clinicians to implement shared decision-making, early diagnosis, and effective treatments well. Hence, this review provides a better understanding of IC/BPS by investigating the epidemiology, etiology, pathology, diagnosis, treatment, and fundamental research on IC/BPS based on the recent literature and guidelines, with the goal of inspiring more in-depth studies and improving the prognosis of IC/BPS patients.

## 2. The Epidemiology, Clinical Symptoms, Etiology and Pathology of IC/BPS

The prevalence of IC/BPS varies widely since uniform definitions and methods are lacking, and current evidence estimates a rate between 0.01% and 6.5% [11]. Many studies have demonstrated differences in the prevalence of IC/BPS across different regions and ethnicities. The prevalence among American women ranged from 2.7% to 6.5% [24,25,26], and in Europe, the estimated morbidity is 300/100,000 in Finland [27], 206/100,000 in Austria [28] and 147/100,000 in Boston [29]. In Asia, approximately 261 Korean women for every 100,000 suffer from IC/BPS [26], and the Japanese population has a similar rate of 0.027% [30]. In contrast, the incidence rate of the disease in China is relatively low, with only 21.8–100 in 100,000 developing IC/BPS [26,31]. The main symptom of IC/BPS is pelvic pain with or without urinary symptoms, and the clinical pattern among young patients is dominated by urgency, frequency, dysuria, dyspareunia, and external genital pain, while that of elderly patients is dominated by nocturia, urinary incontinence, and Hunner lesions [2,32]. In addition, IC/BPS is more common in women than men, with a female-to-male ratio in incidence rates of approximately five to one [33]. Although rarely lethal, IC/BPS is a disruptive condition impacting daily activities, physical health, psychosocial functioning, and quality of life [29]. It is difficult to estimate the socioeconomic burden of IC/BPS accurately, and it deserves more attention regarding the economic burden on individuals. Average annual healthcare costs following the diagnosis of IC/BPS are 2.0 to 2.4 times higher than in age-matched controls [34]. Furthermore, the symptoms of IC/BPS may affect the working hours and job performance of patients, resulting in a particularly significant indirect cost of the disease. 

The etiology of IC/BPS is not fully understood thus far; however, considerable evidence has shown that it involves a complex interplay of neurological, endocrine, immune and other mechanisms [11,35,36]. Among the numerous underlying causes explored in recent years, the following factors have been widely recognized: neurogenic inflammation, infection, autoimmunity, mast cell activation, the defection of glycosaminoglycan (GAG), and the permeability of the bladder epithelium [11,35,36,37,38]. Moreover, IC/BPS may be a systemic disease, and it has been reported to be associated with irritable bowel syndrome, vulvodynia, depression, migraine, sicca syndrome, systemic lupus erythematosus, allergy, and asthma [39,40,41]. Similarly, the pathological mechanism of IC/BPS is undefined. Hunner lesions represent the most characteristic findings, but only a fraction of patients exhibit them, and patients’ diagnoses are divided into Hunner-type interstitial cystitis (HIC) and NHIC based on the presence of Hunner lesions [42]. HIC are characterized by epithelial denudation, which includes increased vessels, obvious edema, and scattered hemorrhage [43]. Severe inflammation of the whole bladder is also identified in patients with HIC, which involves the subepithelial infiltration of lymphocytes, neutrophils and eosinophil granulocytes, macrophages, mast cells, and plasma cells [44]. Moreover, NHIC includes scattered infiltrations of inflammatory cells and moderate-to-dense subepithelial fibrosis [11]. Regarding the classification of IC/BPS, the European Society for the Study of IC/BPS (ESSIC) proposed that IC/BPS could be typed based on cystoscopy after hydrodistension of the bladder and bladder biopsies. The basis for a typing scheme is based on whether any hemorrhagic spots and Hunner lesions are observed via cystoscopy or any inflammatory and fibrotic lesions are observed via biopsy [45].

## 3. Diagnosis of IC/BPS

Since there are no sensitive and specific biomarkers for the diagnosis of IC/BPS, its diagnosis still poses challenges. Different from that of 30 years ago, the diagnosis of IC/BPS does not rely on strict diagnostic criteria; rather, a diagnosis of exclusion and the recognition of symptoms of IC/BPS are key. We summarize the diagnostic recommendations from several guidelines (AUA; CUA; EAU; the International Consultation on Incontinence—Research Society, ICI-RS; the Japanese Urological Association, JUA; and the Royal College of Obstetricians and Gynaecologists, RCOG) and recent studies.

### 3.1. History and Assessment Scale

A detailed history is vital for obtaining a diagnosis. As mentioned by Pape et al. [46], the content of assessments includes the precise nature of the pain, including its location, radiation, and palliative and aggravating factors. The history of pelvic surgery and other autoimmune diseases is also helpful for diagnosis. In particular, the most common urological symptoms are urinary urgency and frequency, which may be present prior to pain occurrence [47,48]. These symptoms are also found in overactive bladder (OAB), and almost all guidelines highlight the need to differentiate these two diseases. It differs from patients with OAB, who urinate frequently to avoid urinary incontinence; IC/BPS patients quell the pain by urinating [49]. A three-day voiding diary can help to distinguish polyuria and assess the symptom severity of female patients. Inevitably, the diagnosis of IC/BPS also requires the exclusion of prostate and urethral diseases, bladder diseases, genitourinary infections, gynecological diseases, and pelvic floor diseases.

CUA, EAU, ICI-RS, and BJOG recommend symptom scales used as tools to assist diagnosis, and there are five commonly used scales (i.e., the Interstitial Cystitis Symptom Index (ICSI); the Interstitial Cystitis Problem Index (ICPI) [49]; the Wisconsin Interstitial Cystitis scale (UW-IC scale) [50]; the Pain, Urgency, Frequency score (PUF score) [51]; and the Bladder Pain/IC Symptom Score (BPIC-SS) [52]). It should be emphasized that these symptom scales are not highly specific and can only be used as diagnostic aids.

### 3.2. Physical Examination and Laboratory Tests

The clinical signs of IC/BPS patients are nonspecific; nevertheless, suprapubic tenderness and bladder neck point tenderness are prevalent in both sexes. More importantly, other reproductive system diseases, such as prostate disease, can be excluded by physical examination. Laboratory tests mainly include urinalysis and urine culture, and AUA, JUA, CUA, EAU, RCOG, and ICI-RS recommend these tests to exclude other diseases, such as bacterial cystitis, tuberculous cystitis, and vaginitis. Urine cytology is also recommended by JUA, CUA, EAU, and BJOG for patients with hematuria (microscopic or gross), or who have a history of smoking, in order to rule out the presence of urinary malignancy. Antiproliferative factor (APF) appears to be a possible urine biomarker for the diagnosis of IC/BPS; however, its level is easily influenced by other factors [53]. Although many other diagnostic biomarkers have been explored, there are no universally accepted items [54,55,56].

### 3.3. Cystoscopy and Bladder Biopsy

JUA, RCOG, CUA, EAU, BJOG, and ICI-RS recommend cystoscopy as part of the initial evaluation to rule out other diseases. Cystoscopy can not only assist diagnosis but also can differentiate HIC from NHIC, which is helpful for the treatment, considering their different responses to therapy [57]. ESSIC has typed IC/BPS based on cystoscopy and bladder biopsies and histopathologic changes, such as inflammatory infiltrates and epithelial denudation, which may be more easily identified by bladder biopsy. Nevertheless, the pathology of the bladder biopsy only serves to rule out other diseases; it is not recommended for the diagnosis of IC/BPS. 

### 3.4. Other Examinations

Some studies have reported that the urodynamic test could confirm the diagnosis of OAB and IC/BPS [58]. However, no guidelines recommend urodynamic investigation as a routine item, and if there are coexisting NHIC and OAB (and/or stress urinary incontinence and/or voiding dysfunction) that are not responsive to treatment, urodynamic tests may be considered. Imaging tests are used in the differential diagnosis of a disease when a patient is suspected to have other comorbid conditions. In the case of gross or microscopic hematuria, it is necessary to perform imaging tests to rule out other urological diseases. Ackerman et al. [59] and Tyagi P et al. [60] reported that magnetic resonance imaging (MRI) has the potential to be applied in subclassifying patients to IHC and NHIC because these subtypes present differently in pelvic muscle hypertonicity and post-contrast bladder wall T1 values. Another study demonstrated that a high bladder wall signal intensity in diffusion-weighted MRI (DWMRI) helps to verify the presence of IC/BPS [61]. However, the diagnostic value of MRI for IC/BPS needs further confirmation, and guidelines do not recommend MRI routinely. The potassium sensitivity test (PST) is based on the dysfunctional epithelium (GAG layer) hypothesis, which is an intravesical therapy using 0.4 mol/L potassium chloride solution to detect the reaction of sensory nerves, and IC/BPS patients will feel pain due to the increase in their bladder mucosal permeability or sensory neurosis. Nonetheless, Yilmaz et al. [62] found that the sensitivity, specificity, and positive predictive and negative predictive value of PST were 50%, 63.5%, 46.5%, and 66.7%, respectively. As the diagnostic value of PST has not been extensively validated, and it is a generally costly and painful process, PST should not be used in the diagnosis of NHIC.

**Figure 1:**
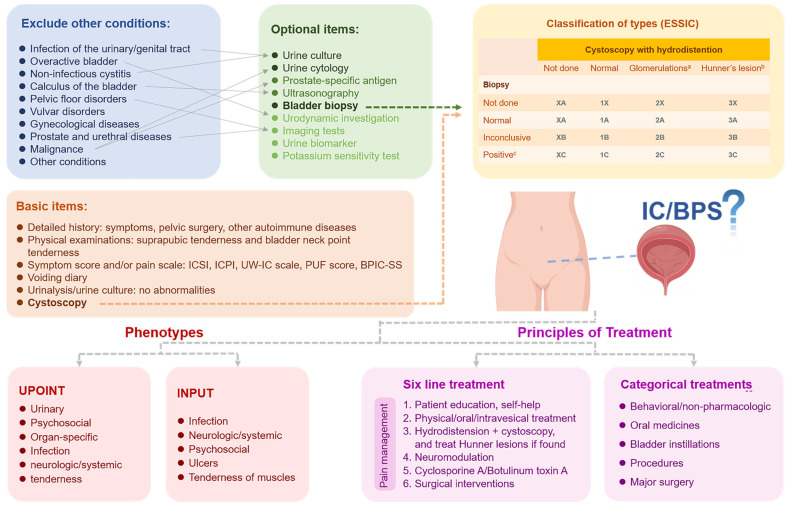
Diagnostic key points, phenotypes classifications, and principles of treatments for interstitial cystitis/bladder pain syndrome (IC/BPS). Conditions in blue square represent the diagnosis of exclusion. Examination in green square represent the optional items that followed by symptoms in the blue square. Items in orange square represent the basic diagnostic points. Yellow square represents the classification of types in IC/BPS diagnosed by cystoscopy and bladder biopsy. Red squares represent two phenotypes in IC/BPS and purple squares represents two different principles of treatment of IC/BPS. ^a^ Cystoscopy: glomerulations grade 2–3; ^b^ With or without glomerulations; ^c^ Histology showing inflammatory infiltrates and/or detrusor mastocytosis and/or granulation tissue and/or intrafascicular fibrosis. IC/BPS, interstitial cystitis/bladder pain syndrome; ESSIC, European Society for the study of IC/BPS; ICSI, Interstitial Cystitis Symptom Index; ICPI, Interstitial Cystitis Problem Index; UW-IC scale, Wisconsin Interstitial Cystitis scale; PUF score, Pain, Urgency, and Frequency score; BPIC-SS, Bladder Pain/IC Symptom Score.

Collectively, IC/BPS is a diagnosis of exclusion, and other known medical diseases must be ruled out before a diagnosis of IC/BPS is confirmed. We provide the diagnostic key points in Figure 1.

## 4. Treatment of IC/BPS

### 4.1. Principles of Treatments

The therapeutic goal is to relieve bladder pain, diminish urgency and frequency, and improve the quality of life (QoL) in patients. However, as the imprecise pathogenic factors and heterogeneity in individuals indicate, the modalities for IC/BPS vary. Individual targeted therapy, therefore, is a necessity. The UPOINT phenotypic classification system developed by Shoskes was recommended by CUA, EAU, and two clinical trials [9,63,64]. Both studies found that approximately 50% of IC/BPS patients treated with corresponding modalities could achieve significant symptomatic improvements. In addition, a novel phenotypic classification system termed INPUT may better meet the demand for personalization than the prior version, although it requires further validation [10] (Figure 1).

AUA has advocated for the six-line treatment since 2011, which consequently boosts the hierarchical standard treatment for IC/BPS [7]. Moreover, in the 2022 version of AUA [1], the panel has transformed this linear system into a more categorical program: from behavioral/non-pharmacologic, oral medicines, bladder instillations, and procedures to major surgery (Figure 1). The update is on account of the emphasis on mutual and in-depth communication between patients and clinicians, personalized factors, and evaluations. Meanwhile, other guidelines also advocate for a similar multimodal treatment system on the basis of shared decision-making to cope with the complexity of IC/BPS [3,4,8,12]. Possible treatment modalities from AUA, CUA, EAU, ICI-RS, JUA, and RCOG are categorically displayed in (Table 1).

### 4.2. Conservative Treatment

It is critical to implement patient education, which could largely facilitate thorough management. The majority of IC/BPS patients were reported to have been experiencing stress, depression, and distress [8,65]. Stress reduction activities, including mindfulness [66], meditation [11], cognitive behavioral therapy [67], and support from family and online networks should be encouraged. In addition, around 90% of IC/BPS patients suffer from symptomatic exacerbations after the increased consumption of coffee, wine, beer, tomatoes, sweeteners, acidic beverages, and spicy foods [68,69,70]. Alternatively, leafy greens, milk, rice, and meat are unlikely to cause the flare-up of these irritative symptoms. Therefore, adjustments to diet should be preferentially encouraged among patients [3,8,11]. 

Massage [71], pelvic trigger point injections [72], and myofascial therapy [73,74] might partially smooth the muscles, as well as relieve pain (AUA, Grade A) [2]. Notably, some physical therapies (e.g., Kegel exercises) may worsen the symptoms in IC/BPS patients [1,2,7]. Moreover, fostering the habit of regular and scheduled voiding contributes to the improvement of bladder sensation and capacity, which was corroborated by Chaiken’s study [75]. 

### 4.3. Pharmaceutical treatment

Amitriptyline possesses the features of being anticholinergic, antihistamine, and sensitivity reducing [76] and is supported by strong recommendations among IC/BPS patients in various guidelines. Pentosan polysulfate (PPS) is the only oral drug that has been approved by the Food and Drug Administration (FDA) for IC/BPS. Taneja [77] found that PPS was beneficial for IC/BPS patients in five out of seven randomized controlled trials (RCTs). Attention should be paid to the risk of macular damage, vision-related injuries, gastrointestinal symptoms, and alopecia when taking PPS on a long-term basis. However, in a large double-blindedRCT, researchers found that PPS with a dosage of 100 mg, once or three times a day, was inefficacious compared to a placebo in the treatment of IC/BPS [78]. Based on this effect and the possible adverse effects, PPS was not recommended by RCOG [12]. Similarly, hydroxyzine as an antihistamine was recommended by EAU and CUA, while it was not recommended by RCOG due to its non-superiority to a placebo in Sant’s study [3,12,79]. Conversely, AUA, CUA, EAU, and RCOG all recommend another antihistamine, cimetidine, in the treatment of IC/BPS (all Grade B). Cyclosporin A is, to some extent, favored by AUA, CUA, EAU, and JUA, but it is not recommended by RCOG due to its short-lasting effect [12]. Additionally, an oral formulation of hyaluronic acid may effectively prevent urinary symptoms after intravesical chemotherapy [80].

Chronic pain greatly influences QoL in IC/BPS patients, and the assessment of pain is a key indicator of therapeutic efficacy. Analgesics are an indispensable part of pain management, while they are weakly recommended in the ICI-RS due to the low evidence and potential side effects. A relatively low level of evidence was also found in clinical treatment with oxybutynin, gabapentin, sildenafil, L-Arginine, misoprostol, and corticosteroids. 

### 4.4. Intravesical Treatment

Intravesical treatments could achieve the better absorption of agents for better bioavailability, although they carry a higher risk of urethral injuries and infections than oral drugs. By blocking nerves in the bladder wall, intravesical lidocaine with sodium bicarbonate for intravesical instillations was recommended by EAU for symptoms in IC/BPS patients (Grade A). Compared to normal saline (190–183 mL, *p* = 0.879), the ability to expand the maximal cystometric capacity (MCC) by using lidocaine (192–261 mL, *p* = 0.005) was also identified in a comparative study [81]. However, it should be noted that IC/BPS patients could only benefit from lidocaine within a limited period, and the present instillation dosages and maintenance intervals should be optimized.

Dimethyl sulfoxide (DMSO) is the only FDA-approved instillation drug for IC/BPS, and its safety (in accordance with the RIMSO-50 standard) was confirmed in healthy adult males in Shimada’s study [82]. In terms of PPS, a meta-analysis including two RCTs showed that intravesical PPS caused no significant increment in the response rate compared to a placebo (RR = 1.09, 95% CI: 0.54–2.22, *p* = 0.80) [83]. However, Lander et al. [84] constructed an intravesical liposomal system that encapsulated PPS, and the results showed that the ICSI and ICPI, as well as the QoL, were significantly improved for two months. Therefore, PPS is still considered optional in some guidelines. 

In a recent network meta-analysis, resiniferatoxin was considered the most effective drug for intravesical instillations in improving ICPI and ICSI. However, limited evidence was available to support its strong recommendation, as is also the case with bacillus Calmette-Guerin. In addition, combination therapies, including hyaluronic acid with chondroitin sulfate [85], PPS, heparin, or hyaluronic acid with lidocaine [86,87,88] could all achieve satisfying outcomes.

### 4.5. Procedures and Major Surgery

AUA [1] recommended an optional low-pressure hydrodistension under cystoscopy within a short period of treatment, and the same was suggested by ESSIC [45,89]. Intravesical instillation with DMSO could improve the efficacy of hydrodistension in HIC [90]. However, high pressure and long-term hydrodistension might cause severe side effects and it is not recommended by AUA, CUA, and JUA.

Botulinum neurotoxin A (BTX-A) was proven effective in improving the symptoms that afflicted IC/BPS patients in several meta-analyses [91,92,93,94]. Furthermore, compared to hydrodistension alone, the sequential therapy of hydrodistension and intravesical injection of BTX-A (200/100 U) was significantly more efficacious in increasing bladder capacity and alleviating pain than hydrodistension alone at three months (all *p* < 0.05), and the success rates in a global response assessment (GRA) were also higher at 24 months (*p* = 0.007) [95]. Therefore, EAU strongly recommends this combination therapy as an option if intravesical instillations fail. However, because BTX-A requires periodic injections, which should be extensively discussed with patients, and has an ambiguous effect on HIC, BTX-A is also weakly recommended in other guidelines.

Sacral neuromodulation was also efficacious in the control of pain and lower urinary tract symptoms in Wang’s study [96]. Interestingly, a recent meta-analysis found that, among patients with CPPS, sacral neuromodulation was more suitable for non-IC/BPS patients than IC/BPS patients [97]. 

Rofeim et al. [98] and Okui et al. [99] reported a success rate of 100.00% and 75.00%, respectively, with the transurethral YAG laser treatment of IC/BPS. The removal of Hunner lesions in less than 25% of the bladder by fulguration was reported to be effective after a median follow-up of 44.8 months [100]. Similar results were displayed in a Korean group [101], focusing on the effect of the cauterization and resection of Hunner lesions. Additionally, Lee et al. [102] reported that 49.2% of patients experienced alleviations of pain and urinary symptoms with a combination therapy involving the resection of Hunner lesions with hydrodistension in 132 IC/BPS patients. Most guidelines recommend fulguration/transurethral resection as a supplementary method for refractory IC/BPS patients, especially HIC.

Major surgeries are the final-line treatment for strictly selected patients with IC/BPS. Specifically, severe bladder fibrosis [99], the presence of Hunner lesions [103,104,105], and reduced bladder capacity [106] are three independent predictors for a better prognosis after radical surgery. Queissert et al. [105] conducted a 14-year follow-up of patients receiving radical surgeries (including augmentation cystoplasty with ileum or ileocecum and supratrigonal cystectomy), and 95.60% of them were satisfied with the improvements. Categorically, total cystectomy and orthotopic neobladder might benefit patients with treatment-refractory IC/BPS more than other radical surgeries [107].

### 4.6. Emerging Treatments

**Figure 2:**
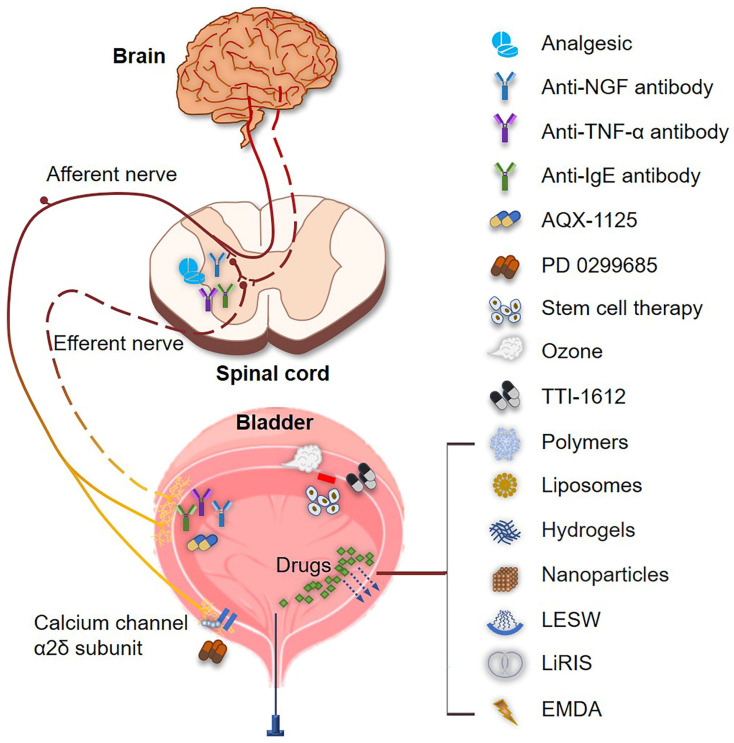
The emerging therapies in IC/BPS. Analgesics down-regulate the neuro-transmitter systems to alleviate the pain; antibodies (including anti-NGF, anti-TNF-α, and anti-IgE antibodies) inhibit the activation of inflammatory factors to improve the symptoms; AQX-1125 inhibits the activation of mast cells to reduce inflammatory reactions; PD-0299685 acts as the calcium (2+) channels α2δ subunit ligand to inhibit the transmission of pain sensation from the bladder wall; stem cells, liposomes, ozone, and TTI-1612 could repair the impaired bladder urothelium; ozone is able to reduce the number of mast cells and leukocytes to inhibit inflammatory reaction; the novel delivery systems, including polymers, liposomes, hydrogels, nanoparticles, LESW, LiRIS, and EMDA, facilitate the penetration and absorption of the loaded drugs into the bladder urothelium (blue arrow). EMDA, electromotive drug administration; IgE, immunoglobulin E; LESW: low energy shock wave; LiRIS: lidocaine-releasing intravesical system; NGF, nerve growth factor; TNF-α, tumor necrosis factor-alpha.

In addition to the present treatment modalities, more innovative therapies, including monoclonal antibodies, novel delivery systems, and other therapeutic modalities, are emerging for the treatment of IC/BPS. Table 2 displays the ongoing clinical studies on the treatment of IC/BPS. (National Library of Medicine (NLM) (accessed on 24 September 2022); available online: https://www.ClinicalTrials.gov). And Figure 2 shows the overview of the emerging therapies in IC/BPS.

#### 4.6.1. Monoclonal Antibodies

Monoclonal antibodies are now gradually being applied in clinical studies in IC/BPS patients. Elevated levels of serum tumor necrosis factor-alpha (TNF-α) and other proinflammatory factors are considered to be correlated with the pathogenesis of IC/BPS [108]. The anti-TNF-α monoclonal antibody adalimumab was proven effective in a phase 3 RCT [18]. Moderate and severe IC/BPS patients were subcutaneously injected with adalimumab 40 mg every two weeks for 12 weeks, and they displayed a significant improvement in ICSI (*p* = 0.0011) and ICPI (*p* = 0.0002), while the difference in all outcomes was insignificant between the two groups. Another anti-TNF-α antibody termed Certolizumab Pegol was identified in a meta-analysis [19]. 

Other drugs, including anti-nerve growth factor (NGF) antibodies, Tanezumab [20], Fulranumab (JNJ-42160443) [21], and anti-IgE antibody Omalizumab [109], were also reported in the literature. However, safety evaluations and long-term follow-ups are still required to confirm the efficacy of antibodies.

#### 4.6.2. Novel Delivery Systems

Given that the efficacy of traditional intravesical instillation is restricted by the voiding of urine and limited penetration into the urothelium, novel delivery methods including electromotive drug administration (EMDA) [110,111], stereolithography (SLA) three-dimensional printing indwelling bladder devices [13], lidocaine-releasing intravesical systems (LiRIS) [14], and nano-based carriers [15,16] could boost the pharmacokinetics in IC/BPS. Specifically, nano-based carriers are of current interest. Structured as phospholipid bilayers, liposomes could carry various materials into the cell through endocytosis [112]. Hence, the combination of liposomes and BTX-A was proven effective in a prospective, multicenter RCT [113]. Researchers encapsulated BTX-A into lipotoxin for intravesical instillations in IC/BPS patients, and statistically significant reductions were obtained in ICIS (4.00 ± 4.28), ICPI (3.35 ± 5.11) and visual analog scale (VAS) (1.64 ± 2.52). Moreover, NGF antisense oligonucleotide [114] and tacrolimus [115] encapsulated with liposomes might also be effective. In addition, the characteristics of being anti-inflammatory and beneficial for bladder urothelium repair also facilitate the sole utilization of liposomes in IC/BPS [116]. 

Additionally, hydrogels, such as TC-3 gel mixed with BTX-A [117], silk-elastin-like protein polymer-based semi-synthetic glycosaminoglycan ethers [118], heparin-loaded Poloxamer 407 hydrogel [119], trimethoprim encapsulated into chitosan-thioglycolic acid nanoparticles [120], polymeric compositions combined with lidocaine and oxybutynin (TRG-042) [121], and low energy shock waves solely or jointly combined with BTX-A [122] also constitute novel delivery systems for the better bioavailability of loaded drugs.

#### 4.6.3. Analgesics 

By down-regulating the neurotransmitter system, cannabinoids were found to represent a promising strategy, as reported in a mouse model of IC/BPS [123,124]. The lack of clinical studies has restricted their recommendation in AUA. Other potential analgesics, including ketorolac tromethamine, naltrexone, oxycodone, naloxone, and ASP3652, also require further research on their effectiveness and safety. 

#### 4.6.4. Stem Cell Therapies

One of the characteristics of IC/BPS is the impaired bladder urothelium and glycosaminoglycan. Furthermore, stem cells—for example, mesenchymal stem cells (MSCs)—might amend the lesions and improve the symptoms [22]. Shin et al. [23] reported the 12-month follow-up of three IC/BPS patients treated with MR-MC-01, as well as MSCs derived from human embryonic stem cells (hESCs). VAS and their lesions were greatly improved, and one lesion in the first patient even became unidentifiable under cystoscopy. Considering the possible immune activation and tumorigenicity of stem cell therapy, MSC-derived extracellular vesicles (MSC-EVs) might resolve this problem. Further studies are needed to assess this potential therapeutic strategy [125,126].

#### 4.6.5. Other Therapies

In a mouse model, oxytocin was found to attenuate hypersensitivity and alleviate pain and stress; thus, it could act as a promising agent for patients with IC/BPS [127]. Similarly, MN-001 was described as an antagonistic leukotriene inhibitor for phosphodiesterase IV [128], and doses of 30 and 50 mg/kg MN-001 in mice were effective in protecting the bladder from hyperactivity. 

In recent studies, the interaction of dysbiosis of urinary microbiota and the host was found to induce the activation of neurons on the bladder wall [129]. Hence, probiotics may theoretically improve urinary symptoms and pain sensation, although further studies are needed. 

PD 0299685 is a calcium channel α2δ subunit ligand, and a double-blind phase II RCT showed that a daily dose of 60 mg of PD 0299685 was sufficient to produce a significant reduction in pain severity compared with a placebo at week 12 [130]. However, no ICSI and urinary symptomatic improvements were identified. Moreover, SH2-containing inositol-5′-phosphatase 1 (SHIP1) protein could inhibit the phosphoinositide-3-kinase (PI3K) pathway and reduce the cascade of inflammation. AQX-1125 is an activator of the SHIP pathway, with the features of inhibiting the activation of mast cells for inflammation; therefore, AQX-1125 could be a potential drug for IC/BPS [131,132]. However, Nickel et al. [131] found that IC/BPS patients treated daily with 100 or 200 mg AQX-1125 demonstrated no difference in ICSI, BPIC-SS, frequency, and pain improvements. In Bayrak’s study [133], ozone could reduce the number of mast cells and leukocytes, expedite angiogenesis, and boost collagen accumulation and fibroblastic proliferation to repair the defects of the urothelium in a chemical cystitis rabbit model. However, its effect on the IC is still unclear. TTI-1612, a soluble heparin-binding epidermal growth factor (HB-EGF)-like growth factor, was able to antagonize APF, which have been identified as one of the pathogenic factors for IC/BPS [134]. Therefore, this could be a potential therapeutic strategy for IC/BPS [135]. However, these novel therapeutic strategies require further investigations.

## 5. The Fundamental Research of IC/BPS

The etiology and pathophysiological changes of IC/BPS remain elusive. Here, we present the animal and cell models related to IC/BPS from recent studies to illustrate the diversity. Moreover, the research on the mechanisms of urinary symptoms, pain sensation, and bladder wall fibrosis, as well as the potential targets of IC/BPS, is discussed.

### 5.1. Animal and Cell Models Related to IC/BPS

#### 5.1.1. Animal Models

**Figure 3:**
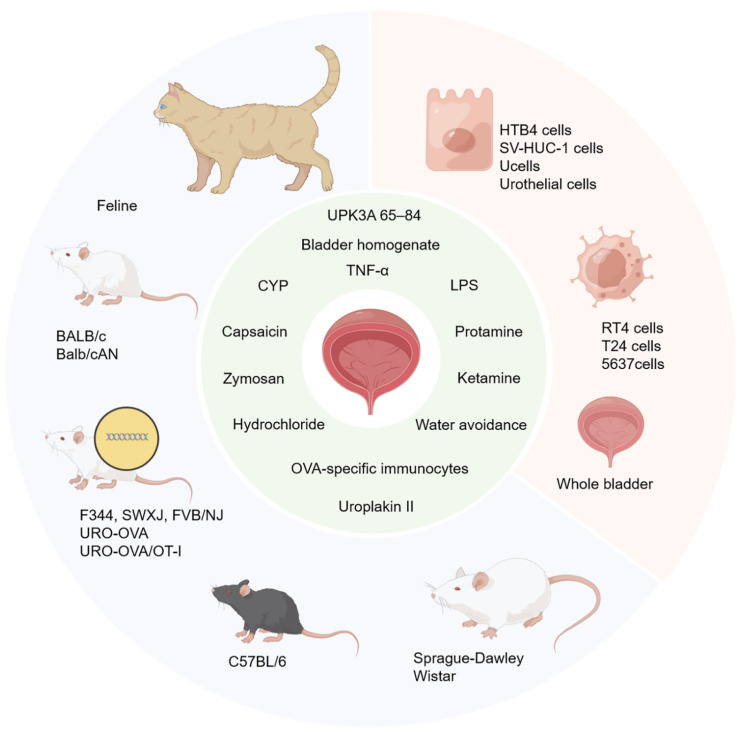
Animal and cell models related to IC/BPS. Feline, mice (including BALB/c and Balb/cAN), transgenic mice (F344, SWXJ, FVB/NJ, URO-OVA and URO-OVA/OT-1), rats (C57BL/6), and transgenic rats (Sprague-Dawley and Wistar) are the animal models related to IC/BPS. Urothelial cell lines (HTB4 cells, SV-HUC-1 cells, Ucells and Urothelial cells) and cancer cell lines (RT4 cells, T24 cells and 5637 cells) and the whole bladder are the cell models related to IC/BPS. UPK3A 65–84, bladder homogenate, CYP, LPS, TNF-α, capsaicin, zymosan, hydrochloride, protamine, ketamine, water avoidance, OVA-specific immunocytes, and Uroplakin II are the stimulants which can induce IC/BPS in animals and cells. CYP, cyclophosphamide; LPS, lipopolysaccharide; TNF-α, tumor necrosis factor-alpha.

To date, many studies have tried to construct animal models of IC/BPS, which are essentially induced by instillation or injection with acids, specific oils, cyclophosphamide, lipopolysaccharide (LPS), and other toxic ingredients [123,136,137,138,139,140,141]. The IC/BPS animal models mimic some specific phenotypes of dysregulation and, thereafter, provide a better understanding of the mechanism of IC/BPS. Table 3 and Figure 3 displays the animal models that are related to IC/BPS.

The majority of animal models are generated from bladder-based phenotypes. Ness et al. [142] constructed a model of neonatal bladder inflammation (NBI) induced by zymosan in a female rat. After intravesical stimulation, footshock stress, and other irritants, rats with NBI experienced increased vigor of abdominal contractions in response to urinary bladder distension, pelvic floor muscle sensitivity, and an elevated level of anxiety, which were symptomatically correlated with IC/BPS patients. Followed by the instillation of cyclophosphamide, the bladder would experience the impairment of the urothelium, hemorrhage, and the cascade activation of mast cells and leukocytes and, consequently, the emergence of lower urinary symptoms and pain sensation [140,143]. However, it should be noted that inflammation induced by cyclophosphamide would be more similar to hemorrhagic cystitis in which lesion phenotypes accounted for most of them [139]. Moreover, the same applies to chemical cystitis caused by acidic agents. In terms of the species, Chen et al. [141] pointed out that C57BL/6J mice induced by LPS showed marked fluctuations in bladder peak pressure, as well as shortened intercontraction intervals, and the treatment was more suitable for IC/BPS than FVB/NJ. 

Psychological/physical stressor models, as well as complicated pathogenic models, are also included within animal models [136,144]. A water avoidance stress type integrated the characteristics of increased frequency, heightened stress responsiveness, and bladder hyperalgesia due to a loss of umbrella cells [136,145,146]. Westropp et al. [147], Buffington et al. [148], and Birder et al. [149] found that the feline IC/BPS model could naturally reproduce many features similar to humans, including the increased levels of some catecholamines and metabolites. On the other hand, IC/BPS could also derive from complicated pathogens present outside the bladder. In particular, experimental autoimmune cystitis (EAC) animal models, as one type of complicated pathogenic model, has also been utilized in the investigation of HIC for a long time. Generally, EAC animal models can be roughly divided into types derived from the induction of bladder homogenate and urothelial antigens, spontaneous types, and transgenic types [137]. Multiple strains of mice have been used for the construction of IC/BPS with the supernatant of bladder homogenate, and considerable efficacy was demonstrated through corresponding treatments [150,151,152]. Autoimmunization by the subcutaneous injection of uroplakin II in rats could cause more obvious manifestations with decreased intercontraction intervals, bladder urothelium barrier impairments, and inflammation than other drugs in Song’s study [153]. A similar immunogenic peptide, UPK3A 65–84, would also give rise to the production of interferon γ (IFN-γ) and interleukin-2 (IL-2) from the activation of CD4 + T cells in the epithelium [154]. Akiyama [155] injected antigen ovalbumin (OVA) from the chicken into mice to collect OVA-specific immunocytes, which were then subcutaneously injected into the URO-OVA mice to produce an OVA-related IC/BPS model. Elevated levels of mRNA expression of TNF-α and IFN-γ were observed in the bladder tissue of this transgenic model. Interestingly, a similar transgenic model was also successfully developed in URO-OVA/OT-I mice. They did not only exhibit pelvic pain and irritative urinary symptoms after being treated with LPS but also suffered from chronic inflammation spontaneously at ≥10 weeks of age [137].

Above all, the analogous animal models should be treated with caution, as most of the models are female-specific and, therefore, might ignore the possible pathophysiological changes in male animals.

#### 5.1.2. Cell Models

Researchers have also attempted to construct cell models in vitro (Table 4). Shao et al. [143] applied LPS and adenosine triphosphate (ATP) to human urothelium SV-HUC-1 cells, and flow cytometry analysis demonstrated that, compared to cells in the control group, apoptosis rates were higher in the experimental group. Increased trans-epithelial permeability was found in the cultivation of human HTB4 cells induced by TNF-α [134]. Similarly, after the treatment of 10 ng/mL TNF-α in SV-HUC-1 cells, the Smad pathway was strongly activated, with the upregulated expression of Smad2 and Slug [156]. Moreover, the increased collagen I and pro-fibrosis cytokines in SV-HUC-1 cells resembled the process of mesenchymal transition (EMT) and fibrogenesis in IC/BPS.

Cells from pigs [157] and rats [158] also contributed to the development of a cell model of IC/BPS. Interestingly, Rapp et al. [159] incubated the whole rat bladder and treated it with capsaicin and ATP, and BTX-A could reduce the release of calcitonin gene-related peptide (CGRP) from the nerve terminals, which is an indicator of symptoms. Furthermore, IC/BPS cell models could also originate from cancer cells. For example, human carcinoma epithelial RT4 cells and T24 cells were treated with TNF-α to mimic the inflammatory condition in IC/BPS [160,161]. Ketamine has been successfully used as a stimulus to induce dysregulation in SV-HCU-1, RT4, and 5637 cell lines [162]. However, the differences between the characteristics of malignant cells compared to benign IC cells should be treated with caution.

**Table 4:** Cell models of IC/BPS in recent studies.

Species	Cell Types	Stimulants	Refs.
Homo Sapiens	SV-HUC-1	LPS and ATP	[140]
Homo Sapiens	SV-HUC-1	TNF-α	[153]
Homo Sapiens	HTB4	TNF-α	[131]
Homo Sapiens	SV-HUC-1	Ketamine	[159]
Sus scrofa	Ucells	Protamine	[154]
Rattus norvegicus	Urothelial cells	Toxic factors	[155]
Homo Sapiens	RT4	TNF-α	[157]
Homo Sapiens	T24	TNF-α	[158]
Homo Sapiens	RT4	Ketamine	[159]
Homo Sapiens	5637	Ketamine	[159]
Rattus norvegicus	Whole bladder	Capsaicin and ATP	[156]

ATP, adenosine triphosphate; LPS, lipopolysaccharide; TNF-α, tumor necrosis factor-alpha.

### 5.2. The Research on Urinary Symptoms in IC/BPS

The evident urinary symptoms in IC/BPS mainly resemble the manifestations of OAB. Physiologically, IC/BPS causes a reduction in bladder capacity and voiding threshold pressures, as well as the onset of pelvic pain [163]. The destruction of the bladder barrier contributes to the production and flare-up of symptomatic changes. One hypothesis is that chronic inflammation would activate mast cells to release inflammatory cytokines, which amplifies the neuroinflammatory responses by increasing trans-urothelial permeability [134,139]. This alteration in the bladder wall prompts the influx of potassium ions and results in neural hypersensitivity and hyperalgesia, which are clinically manifested as urinary urgency, frequency and pain [163,164]. The effect of MSCs on the urinary symptoms of IC/BPS reciprocally prove that the repair of the bladder wall and the reduction of mast cell infiltration are mediators for the improvements.

Another factor that contributes to the induction of urinary symptoms lies in the fibrosis of the bladder wall (see Section 5.4). Decreased bladder capacity affected by the fibrotic wall would restore less urine and therefore exacerbate the urinary frequency and nocturia [164].

### 5.3. The Research on Pain Sensation in IC/BPS

The afferent nerves on the bladder wall consist of Aδ fibers and C fibers. Aδ fibers receive signals from bladder contraction and expansion, while C fibers respond to thermal changes and chemical and pain stimuli [165]. Molecular sensors on the afferent nerve, including transient receptor potential A1 (TRPA1) [166], TRPV1 [167], and transient receptor potential cation channel subfamily M member-3 (TRPM3) channel [168], have been identified as mediators for the conveyance of pain signals. In addition, the TRPM3 channel also acts as a thermal sensor, causing thermal hypersensitivity in humans [169]. Recent studies demonstrated that TRPA1, TRPV1, and TRPM3 were involved in the development of chronic pain in IC/BPS, and the inhibition of these channels was proven effective in alleviating the severity of pain [165,170]. Similarly, from the results of the von Frey filament experiment in Chen’s study [171,172], neuregulin-1-ErbB and Notch1 signaling might boost the activation of microglia in cyclophosphamide-induced cystitis and, therefore, could be two potential targets for the treatment of allodynia.

In addition, the elevated mRNA expression of substance P precursor, one of the pain mediators, was observed in the inflammatory bladder [138]. Other factors, including brain-derived neurotrophic factor (BDNF) through BDNF-TrkB-p38/JNK signaling [173], IL-33 through the IL-33-mast cell-dependent axis [174], and chemokine (C-C motif) ligand 2 (CCL2) though the accumulation of mast cells [174], could aggravate neuroinflammation and hyperalgesia. However, in a LL-37 IC/BPS model in Jia’s study [175], the degree of pain was independent of the extent of inflammation in the bladder wall. This is supported by the phenomenon wherein patients with NHIC could also suffer from intermittent and chronic pain.

### 5.4. The Research on Bladder Wall Fibrosis in IC/BPS

One important pathological characteristic of IC/BPS lies in the fibrosis of the bladder [164]. A prospective cohort study collected 100 IC/BPS patients, and HIC patients were characterized by diffuse and focal bladder thickening (*p* < 0.001) [17]. Jin et al. [156] conducted an experiment in vitro by stimulating human epithelial SV-HUC-1 cells with TNF-α, and minor inflammation caused by TNF-α could promote the accumulation of collagen, which constituted pro-fibrogenesis expression. Genetically, down-regulating WNT11 would promote EMT activation and bladder fibrosis, especially in patients with NHIC [176,177]. Therefore, WNT11 might be a potential marker for predicting NHIC. Moreover, YKL-40 is another possible indicator for the assessment of bladder fibrosis [178]. 

In addition, microRNAs (miRNA) have been reported to be correlated with inflammatory reactions and the growth and fibrosis of the bladder wall. In recent studies, miRNA-495 [179] was found to inhibit bladder fibrosis through the Janus kinase-signal transducer of activation (JAK-STAT) pathwayand miRNA-139-5p targeting LPAR4 could reduce bladder EMT and fibrosis through the phosphatidylinositol-3-kinase/Akt (PI3K/Akt) pathway [180]. On the contrary, a miRNA-132 [181] mimic could increase the expression of IFN-γ, TNF-α, intercellular adhesion molecule-1 (ICAM-1), and collagens I and III in rat IC/BPS models, and therefore miRNA-132 was found to be a risk factor for the promotion of detrusor fibrosis. Better performance of urodynamic parameters and reductions in corresponding inflammatory and fibrosis-related factors were promoted by the inhibitor of miRNA-132.

## 6. Conclusions

We have summarized the advancements in the diagnosis, treatment, and fundamental research of IC/BPS and set new directions for clinical and scientific studies and investigations. Due to the complexity of IC/BPS, treatment is no easy task. A promising trend is multimodal treatment targeting identified phenotypes. Integrating deep machine learning and other interdisciplinary technologies could boost the efficacy of diagnosis and treatment. Moreover, significant potential lies in identifying markers in patients who may develop vulnerability to IC/BPS symptoms. After early diagnosis, a comprehensive and periodic evaluation of the systematical states and the symptoms of IC/BPS patients, with constant and in-depth communication with patients, would lead to optimized therapeutic modalities. 

## Figures and Tables

**Table 1:** The present treatments for IC/BPS based on grade of recommendations.

Category	A	B	C	D	CP/EO	Not Recommend
**Conservative therapies**						
Education	CUA	EAU *, JUA	ICI-RS *	RCOG	AUA	-
Physiotherapy	AUA	CUA, EAU, RCOG	ICI-RS, JUA	-	-	-
Stress reduction	-	CUA, EAU, JUA	ICI-RS	RCOG	AUA	-
Diet treatment	-	CUA, JUA	EAU, ICI-RS	RCOG	AUA	-
Bladder training	-	CUA, EAU	-	-	AUA	-
Acupuncture	-	CUA	JUA	-	-	-
**Oral treatments**						
Amitriptyline	EAU	AUA, CUA, ICI-RS, JUA, RCOG	-	-	-	-
Hydroxyzine	EAU	CUA	AUA, JUA	ICI-RS	-	RCOG
Pentosan polysulfate	EAU	AUA, JUA	-	CUA, ICI-RS	-	ROCG
Cimetidine	-	AUA, CUA, EAU, RCOG	ICI-RS, JUA	-	-	-
Cyclosporine A	-	-	AUA, CUA, ICI-RS, JUA	-	-	RCOG
Oxybutynin	-	-	CUA, EAU, JUA	ICI-RS	-	-
Gabapentin	-	-	CUA, ICI-RS, JUA	-	-	-
Quercetin	-	-	ICI-RS	ICI-RS	-	-
Analgesics	-	-	ICI-RS, JUA	-	AUA, RCOG	-
Sildenafil	-	-	CUA, JUA			
L-Arginine	-	-	EAU	JUA	-	-
Misoprostol	-	-	EAU	-	-	-
Corticosteroids	-	-	JUA	-	-	AUA, EAU
Antibiotics	-	-	-	ICI-RS, JUA	-	AUA, JUA
Duloxetine	-	-	-	JUA	-	EAU
**Intravesical therapies**						
Lidocaine	EAU	AUA, CUA, RCOG	ICI-RS, JUA	-	-	-
Dimethyl sulfoxide	-	CUA, ICI-RS, JUA	AUA, RCOG	-	-	-
Pentosan polysulfate	EAU	-	CUA, JUA	ICI-RS	-	-
Heparin	-	ICI-RS	AUA, CUA, EAU, JUA	RCOG	-	-
Hyaluronic acid	-	EAU, RCOG	CUA, JUA	ICI-RS	-	-
Chondroitin sulfate	-	EAU	JUA	CUA, ICI-RS, RCOG	-	-
Oxybutynin	-	-	CUA, EAU, JUA	-	-	-
Corticosteroids	-	-	EAU, JUA	-	-	-
Resiniferatoxin	-	-	CUA, JUA	-	-	CUA, ICI-RS, RCOG
BCG	-	AUA	-	JUA	-	CUA, EAU, ICI-RS, RCOG
**Procedures**						
BTX-A and HD	EAU	-	AUA	ICI-RS	-	-
Neuromodulation	-	EAU, JUA	AUA, CUA, ICI-RS, JUA	RCOG	-	-
Fulguration/ablation	-	CUA, JUA	AUA, EAU, ICI-RS	-	RCOG	-
BTX-A injection	-	JUA, RCOG	AUA, AUA	-	-	-
Hydrodistension	-	JUA	AUA, CUA, EAU, ICI-RS, JUA	-	-	-
Triamcinolone	-	-	AUA	-	-	-
Hyperbaric oxygen	-	-	CUA, JUA	-	-	EAU
**Major surgery**						
Radical surgries	EAU	-	AUA, CUA, ICI-RS, JUA	RCOG	-	-

BCG: Bacillus Calmette-Guerin; BTX-A: Botulinum toxin A; CP: Clinical Principle; CUA: Canadian urological association; EAU: European Association of Urology; EP, Expert Opinion; HD, Hydrodistension; ICI-RS, the International Consultation on Incontinence-Research Society; JUA, Japanese urological association; RCOG, Royal College of Obstetricians and Gynaecologists. * The evidence from EAU, ICI-RS is standardized by the Oxford Centre for Evidence-Based Medicine: Levels of Evidence (March 2009) (Levels of evidence—Centre for Evidence-Based Medicine (CEBM), University of Oxford).

**Table 2:** The ongoing clinical studies on the treatment of IC/BPS.

Therapy	Identifier	Country	Phase	Current Status
**Monoclonal antibody**				
Adalimumab	NCT01295814	USA	III	Completed
Certolizumab pegol	NCT02497976	USA	III	Completed
Omalizumab	NCT01294878	Italy	III	Completed
Tanezumab	NCT01030640	USA	I	Completed
PF-04383119	NCT00601484	USA	II	Completed
Fulranumab	NCT01060254	Multinational	II	Terminated
ASP6294	NCT03282318	Multinational	II	Completed
**Delivery-related system**				
LiRIS	NCT02411110	Multinational	II	Completed
LiRIS	NCT02395042	Multinational	II	Completed
LiRIS	NCT01879683	USA	I	Completed
LiRIS	NCT01559961	Canada	I	Completed
LP-08	NCT01393223	USA	II	Completed
Liposomes	NCT01083979	USA	-	Completed
Liposomes	NCT01731470	USA	-	Completed
LESW	NCT03619486	China	-	Completed
LESW+BTX-A	NCT05275647	China	II	Recruiting
LiESWT	NCT05337813	China	-	Recruiting
**Analgesics**				
Ketorolac Tromethamine	NCT02000401	USA	IV	Completed
Naltrexone	NCT04313972	USA	IV	Recruiting
Naltrexone	NCT04450316	USA	II	Recruiting
ASP3652	NCT01613586	Multinational	II	Completed
Oxycodone naloxone	NCT01197261	Multinational	II	Completed
**Stem cell therapy**				
MR-MC-01	NCT04610359	Korea	I	Recruiting
AlloRx	NCT05147779	Antigua and Barbuda	I	Recruiting
**Other therapies**				
Oxytocin	NCT00919802	USA	IV	Completed
PD 0299685	NCT00739739	Multinational	II	Completed
AQX-1125	NCT01882543	Multinational	II	Completed
MN-001	NCT00295854	USA	II	Completed
Ozone	NCT04789135	Brazil	II	Active, not recruiting
TTI-1612	NCT01559961	Canada	I	Completed

LESW: Low energy shock wave; LiESWT: Low-intensity excoporeal shock wave therapy; LiRIS: lidocaine-releasing intravesical system.

**Table 3:** Animal models of IC/BPS in recent studies.

Species	Strain	Stimulants	Refs.
Rattus norvegicus	Sprague-Dawley	Zymosan	[139]
Rattus norvegicus	F344	Hydrochloride	[136]
Rattus norvegicus	Sprague-Dawley	CYP	[137]
Mus musculus	C57BL/6	CYP	[140]
Mus musculus	C57BL/6	LPS	[138]
Mus musculus	FVB/NJ	LPS	[138]
Rattus norvegicus	Wistar	Water avoidance	[142,143]
Felis catus	-	-	[144,145,146]
Mus musculus	C57BL/6	Bladder homogenate	[147]
Mus musculus	SWXJ	Bladder homogenate	[148]
Mus musculus	Balb/cAN	Bladder homogenate	[149]
Rattus norvegicus	Sprague-Dawley	Uroplakin II	[150]
Mus musculus	BALB/c	UPK3A 65–84	[151]
Mus musculus	URO-OVA	OVA-specific immunocytes	[152]
Mus musculus	URO-OVA/OT-I	-	[134]
Mus musculus	URO-OVA/OT-I	LPS	[135]

CYP, cyclophosphamide; LPS, lipopolysaccharide.
